# Pathological Notes

**Published:** 1880-04

**Authors:** 


### PATHOLOGICAL NOTES.

---

10. TUBERCULOUS MENINGITIS IN A SUN-BEAR.—Through Mr. Conklin, of the Zoölogical Department, Central Park, we had the opportunity of examining the brain of a sun-bear (*Helarctos malayanus*) which, after presenting symptoms of stupor and an intense uncontrollable diarrhœa, died on the 16th of March. The brain and cord were the seat of an extreme arterial hyperæmia. The cerebral tissues themselves were firm. Filling out the extensive arachoid spaces, behind the pituitary body, in front of the chiasm, and on the inside of the temporal lobes was a continuous organized exudate of coalulated lymph, which extended into the sylvian fissure; scattered over the contiguous pia were tubercles varying from the microscopic and transparent perivascular nodules to large yellow or white masses of the size of a pin's head. These were mostly scattered over the base of the brain, involving the pia of the region in front of and behind the pons. The sylvian fissure showed them in the richest profusion; towards the convexity they decreased in number and size, so that to the naked eye this region appeared even healthy. The cerebrospinal fluid was discolored by blood, but otherwise showed no turbidity. There were a few punctiform extravasations under the ependyma of the lateral ventricles.

